# Consequences of migratory distance, habitat distribution and season on the migratory process in a short distance migratory shorebird population

**DOI:** 10.1186/s40462-023-00400-6

**Published:** 2023-07-18

**Authors:** Linus Hedh, Anders Hedenström

**Affiliations:** Department of Biology, Ecology Building, Lund University, Sölvegatan 37, 223 62 Lund, Sweden

**Keywords:** Common ringed plover, Flight durations, Flight performance, Multi-sensor loggers, Migration strategies, Time-minimization, Charadrius hiaticula

## Abstract

**Background:**

The migratory process in birds consists of alternating periods of flight and fueling. Individuals of some populations make few flights and long stopovers, while others make multiple flights between short stopovers. Shorebirds are known for executing marathon flights (jumps), but most populations studied are long distance migrants, often crossing major barriers and thus forced to make long-haul flights. The sub-division of migration in short/medium distance migratory populations, where the total migration distance is shorter than documented non-stop flight capacity and where routes offer more homogenous stopover landscape, is little explored.

**Methods:**

Here we combine data based on conventional light level geolocators and miniaturized multi sensor loggers, comprising acceleration and light sensors, to characterize the migratory routes and migration process for a short/medium distance (~ 1300 to 3000 km) migratory population of common ringed plover (*Charadrius hiaticula*) breeding in southern Sweden. We were specifically interested in the variation in number and duration (total and individual) of flights/stopovers between seasons and in relation to migration distance.

**Results:**

Most stopovers were located along the European Atlantic coast. On average 4.5 flights were made during autumn migration irrespective of migration distance, but in spring the number of flights increased with distance. The equal number of flights in autumn was explained by that most individuals migrating farther performed one longer flight (all but one lasting > 20 h), likely including crossing of the Bay of Biscay. Median duration of single flights was 8.7 h in autumn and 5.5 h in spring, and median stopover duration was ~ 1 day in both seasons. There was a positive relationship between total flight duration and migration distance, but total flight duration was 36% lower in spring compared to autumn.

**Conclusions:**

Our results suggest that when suitable stopovers are abundant common ringed plovers prefer making shorter flights even if longer flights are within the capacity of the species. This behaviour is predicted under both time and energy minimizing strategies, although the variable flight distances suggest a policy of time selected migration. Even if populations using several stopovers seem to be more resilient for environmental change along the route, these results are informative for conservation efforts and for predicting responses to future environmental change.

**Supplementary Information:**

The online version contains supplementary material available at 10.1186/s40462-023-00400-6.

## Introduction

Each year millions of birds conduct seasonal migrations between areas used for reproduction and survival, respectively. Migration is a process typically divided into alternating episodes of directional movement and stopovers, which offer time for recovery and energy (fuel) accumulation for subsequent flight(s) [1]. These episodes are particularly pronounced in long-distance migratory birds, which are able to cover long flight distances and are dependent on large fuel loads to sustain the high energy demands [2, 3]. The sub-division between these episodes throughout the migratory journey may differ between populations (or species) in that some make use of a few long flights between a few longer stopovers, while others divide the migration in multiple flights between many stopovers of short duration [4, 5].

There are several non-mutually exclusive explanations to how and why migration is divided between flights and stopovers. Suggested factors behind the arrangement of stopover/flight lengths include the distribution of suitable stopover sites [5–7], predictable wind patterns [8], physiological constraints related to fuel accumulation and physiological flexibility [9, 10], the need for rest, recovery and sleep [11] and the location of ecological barriers [12–14]. Also, the interplay between migration strategy (i.e. time or energy minimization) and stopover site distribution and quality may influence the sub-division of migration. Two main contrasting strategies are the minimization of energy cost of transport or the overall time of migration, which are associated with different responses of for example departure fuel load to variation in rate of energy (fuel) accumulation and cost (energy and time) of settling at a new stopover site [2, 15]. An energy minimizer is not sensitive to fuel deposition rate and will therefore depart from stopovers with fuel loads only to reach the next stopover, only depending on the search/settling energy cost. In theory, if suitable stopover sites are available everywhere and everything else being equal, this should result in flights of equal step length. In contrast, a time minimizer will respond to varying fuel deposition rates, resulting in varying departure fuel loads, and thus associated flight step lengths [15]. If fueling rate increases or decreases along the route, a time minimizer is expected to progressively increase or decrease the flight distance between stopover sites (as a function of the optimal departure fuel load), respectively [16]. However, if the fueling rate is relatively stable and stopover sites are assumed to be available everywhere along the route, a given number of flights of equal step length are expected [16].

Most current knowledge about division of migratory flights and stopovers comes from populations (or species) that have long total migratory distances (5000 km or more) and/or have to cross major barriers [17, 18]. However, relatively little is known about the division of migration in short to medium distance migratory populations, in which the total migration distance is well within the range of potential non-stop flights documented in other populations (or species) and where no major barriers are present.

The migration of temperate populations of common ringed plovers (hereafter “ringed plover”) offers an interesting system to investigate how short distance migratory populations divide the migration between flights and stopovers in accordance to habitat distribution. These populations, which breed in northern Europe (including the Baltic Sea), mainly winter along the European Atlantic coast, and the migration distance varies between short movements of a few kilometers (mainly British and Irish populations) up to ~ 4000 km in Scandinavian populations [19, 20]. During the non-breeding period ringed plovers can be found along nearly the whole western-European seaboard and the British Isles, which during migration also includes (sub-) Artic breeding populations on their way to or from Africa [21]. The explanation for this apparent omnipresence of ringed plovers is presumably the distribution of large number of suitable intertidal sites (~ 300 km in between), and other suitable habitats (e.g., saltpans and lagoons) located along the coasts [22]. Furthermore, the non-specialized diet of the ringed plover may also extend the set of suitable stopover sites [23].

Here we make use of two data sets to study the migratory process of a short- to medium distance migratory population of ringed plovers breeding in southern Sweden. One data set is based on conventional light-level geolocators and one on novel miniaturized micro data-loggers (MDLs), which apart from a light-level sensor also contain an accelerometer. We used conventional geolocators to describe the population-specific migratory route. The light-data from the MDLs are used to estimate wintering position and estimate migration distance, while the accelerometer data is used to define flight and stopover periods to be able to relate number of flights and flight/stopover durations with migration distance. By combining these methods we are able to describe the migratory process in more detail, as the accelerometer data provides activity patterns in with high time resolution [24, 25].

Individuals of the studied population have been shown to spread out across the entire documented winter range used by temperate breeding ringed plovers, thus exhibiting a marked difference in individual migration distances varying between ~ 1300 and ~ 3000 km [19]. These distances are all within the range of documented non-stop flights of more long-distance migratory populations of this species [26, 27]. Because of the relatively uniform distribution of potential stopover sites along the European coasts and that the ringed plover is a food generalist [23], we hypothesize that ringed plovers breeding in southern Sweden will make several flights during autumn migration, where number of flights is proportional to migration distance. In spring however, ringed plovers migrate relatively early (February to early March) [19, 28], with progressively harsher weather conditions and thus potentially declining fueling rates from south to north. Therefore, during spring migration we hypothesize that ringed plovers will make use of more and shorter flights when compared with autumn, given that a similar route is taken in both seasons. To inform the discussion regarding migration strategy we parameterize two models to predict the number of flights/stopovers for an energy- and time minimizer, respectively, assuming that stopovers are abundantly available along the route.

## Methods

### Field work

We studied plovers breeding on grazed meadows at the southernmost part of Öland (56° 13′ 58″ N, 16° 24′ 40″ N) in southeastern Sweden. Between 2013 and 2017 a total of 53 ringed plovers were caught while incubating and fitted with conventional geolocators (n = 18 in 2013; 12 in 2014; 16 in 2015, 7 in 2016) of the model MK10s (the 2013 batch without ‘stalk’, but thereafter with ‘stalk’) from Migrate Technology Ltd. Loggers were attached with a leg-loop harness made from braded, mist net shelf-string (BTO, The Nunnery, Thetford, Norfolk) [29]. The device (1.6 g) constituted on average 2.4% of individual body mass at capture. Mean body mas (± SD) was 66.4 ± 4 g. Geolocators were retrieved during the following years. A total of 24 geolocators were retrieved (distributed on 20 individuals), of which 8 geolocators contained data on 2 autumn migrations and 1 spring migration. Thus, 32 autumn and 24 spring tracks were available for analyses.

Between 2016 and 2020 a total of 78 multisensory data loggers (MDLs) were deployed (n = 4 in 2016; n = 4 in 2017; n = 19 in 2018; n = 20 in 2019; n = 16 in 2020) on a total of 60 individuals. MDLs were attached using the same procedure as for the geolocators, whereas the device (1.5 g) corresponded to on average 2.3% of the body mass at capture. Mean body mass was 64.3 ± 4 g. In total, 31 MDLs were retrieved and 17 of them (distributed on 15 individuals) contained useful data. Two MDLs recorded for 0.5 years (i.e., covering one autumn migration only), 11 for a full year, 1 for 1.5 years (i.e., 2 autumn and 1 spring migration) and 3 for 2 full years. Thus, the full data set contained information on 21 autumn and 17 spring migrations.

The return rate of tagged birds were closely monitored in the springs of 2019–2021. Average return rate of individuals tagged in 2018–2021 was 68% (range 60–75%), which is lower than the calculated average apparent survival rate (86.6%) of a color ringed population in southwestern Sweden [30]. However, compared to the study in western Sweden searches and trapping efforts were primarily made in one small area (~ 1.5 km radius), instead of over several sites [30]. Thus, even relatively short dispersal distances of individuals between years could go undetected and the true return rate, or apparent survival, of individuals in our study is most likely underestimated rather than an increased mortality rate due to logger effect.

### Hardware and sampling routines of multi-sensor data loggers

The MDLs were custom built and comprise an accelerometer and light sensor [24, 31]. The purpose of the accelerometer is to continuously record activity. Activity was recorded every 5 min at 100 Hz in the vertical Z-axis during 5 consecutive subsampling sessions at 5 s intervals, each lasting 100 ms. The occurrence of activity (0 or 1) in each subsample was summed for each recording, generating an activity score between 0 and 5. Thus, for each hour 12 activity scores (0–5) were recorded that were used to analyze flight activity. A diagram of the sampling routine is shown Additional file 1: Fig. S1.

Activity sampling was pre-programmed to start on 15 July and ran continuously until the logger was removed or the battery was drained of energy. In contrast to conventional geolocators the MDLs only measured light during certain specified periods, lasting up to 20 days in this study. Light measurements were taken as follows: 1–20 August, 1–10 December, 1–10 February, and 21 February to 3 March. This scheme applied to all MDLs with one exception, a logger that belonged to a test version with a different sampling routine for light measurements during migration periods than those used thereafter. These periods were chosen to cover the main migratory and wintering periods. The migratory periods occur from late July to mid-August in autumn and late February to early March in spring [28]. In this study we only use the light-data from the MDLs to estimate winter positions (see *Analysis of light data* section) and thus, we only use light measurements taken in December and early February. For more technical details about sampling routines and the use of accelerometer data see Bäckman et al. [24, 31].

### Analysis of activity data

To identify flight periods based on the accelerometer data, we derived weighted hourly activity scores by calculating the sum of each hourly score multiplied by the number of samples within each hour. Thus, the lowest weighted score becomes 0 (0 × 12) and the highest possible score is 60 (5 × 12) (Additional file 1: Fig. S1). We then identified all hours with the arbitrary chosen weighted activity score of ≥ 30. This allowed us to identify clusters of hours with continuously high activity scores between 3 and 5. In almost all cases these clusters generated sequences of high scores and were almost never found outside the general migratory periods in July–August and February–April [19] (see Additional file 1: Fig. S2 for an example actogram).

Start and end times of the flight were defined around the above defined flight period by subtracting all measurements (5 min periods) falling under a score of 3 (note the difference from weighted score; see Additional file 1: Fig. S1 for example). Specifically, for a flight to be defined as ended zero-scores must be present. Thus, the start and end points of flight periods, and hence flight duration, were defined with 5-min resolution, provided that all scores < 3 and all scores ≥ 3 were recorded in blocks, respectively. All periods between periods of flights were defined as stopovers or winter/breeding site residency, depending on season, and the duration of these was calculated in the same way as the flight periods.

In some cases, particularly at the end of migratory flights, hourly weighted scores could fall below 30. In such cases we exanimated the distribution of the activity scores (0–5) and defined the hour as *in flight* as long as no zero-scores were recorded. If a zero-score was recorded we looked at the following hour to asses if the bird could be considered as having landed by summing the number of zeros between the two hours. If that sum was ≥ 12 (corresponding to ≥ 1 h of inactivity) the bird was considered as landed, but if it did not the bird was considered as having continued flying and the sequence was recorded as one flight. However, when calculating the duration of the flight, all 5 min scores < 3 were omitted from the flight duration. This is because we only can assume that lower scores within flight periods are not associated with landings. One biological reason for why to include lower weighted scores and classify them as *in flight* could for example be descending flights with reduced flapping frequency. This may also occur during mid-flight due to altitude adjustments [32]. Whenever subsampling had partly or fully failed in a given hour associated with a flight period (e.g. only 10/12 samples), these missing subsamples were defined as zeros and excluded when calculating flight duration.

### Analysis of light data

For the conventional geolocators we defined twilight using the R package *TwlGeos* [33] by setting the threshold value to 5 lx, respectively. Annotated twilight events were visually inspected and obvious erroneous events were either corrected (based on the timing of twilight events during the pre- and proceeding days) or removed if the event was considered being caused by e.g. artificial light in the middle of a night. Positions for each geolocator were translated from light measurements to geographical positions using the R package *GeoLight* [34]. In order to find the sun angle corresponding to the set threshold value for each individual geolocator we performed a Hill-Ekstrom calibration for the main stationary period during the non-breeding season [35]. This was done by modeling latitudes against several possible sun elevation angles with 0.1 degrees increments. The sun elevation angle that minimized the error in latitude around the autumn equinox and generated stable latitudinal positions over the course of the wintering period (i.e. no systematic concave or convex shape on the latitude versus time diagram) was selected for each geolocator.

Because light intensity was measured during relatively short periods by the MDLs, Hill-Ekstrom calibration was not possible, and so we set the light threshold value to 2 lx and the sun elevation angles to -6° in all but two loggers (i.e. applied a “Civil twilight calibration”)[36]. Civil twilight calibrations have been shown to generate latitude estimates with less precision and accuracy compared to other methods of calibration [36]. However, estimation of wintering position of three individual ringed plovers, which were tracked with both conventional geolocators (two for more than one year) and MDLs (one year each), indicate that winter position estimations are reasonable similar using the two methods for conventional geolocators and MDLs, respectively (Additional file 1: Fig. S3). The sun angle for the remaining 2 loggers were set to -5°, because the estimated latitudes between the two light measurements periods during winter (December and February) were unrealistically separated latitudinally and position clusters were located in the Atlantic.

We used the derived positions from the conventional light level geolocators only to describe the general population specific migratory route and to calculate a detour index. The general route was simply identified by connecting consecutive stationary periods. We first defined the start and termination of migratory and stationary periods (wintering/breeding and stopover periods), which were primarily identified by visually inspecting longitude plots in combination with generated maps [37]. Geographical positions were subsequently calculated by averaging (and calculating standard deviation) the generated positions within the above defined time periods ≥ 3 days. We excluded 7 autumn tracks for which we could not detect any stopovers lasting ≥ 3 days. These does not necessarily indicate non-stop flights as the average duration between departure and arrival for these was 5.4 days (sd = 2.4 days). Thus, it is more likely that the stops were too short to be detected with the definition used. Because the timing of spring migration largely coincided with the vernal equinox, positions along the migratory route could not be reconstructed for 19 of 23 spring tracks.

For the purpose of this study we only used light data from the MDLs to estimate individual winter positions and estimate migration distance (see below). Thus, light data for this purpose were primarily based on the winter periods of measurements (December and February). However, we also used positions generated from measurements in August and late February as long as activity data showed that the individual bird had arrived at the wintering site or had not yet commenced spring migration. Estimation of winter position followed that of the conventional geolocators (see above). In three loggers, which had recorded activity data for more than one year, the light measurements failed after the first wintering period. For these we assumed the same wintering position recorded the previous year, as we know from re-sightings of color-ringed individuals that ringed plovers usually return to the same wintering area from year to year (L. Hedh, unpublished data).

Migration distance for the MDL’s was defined by using the Harversine formula to calculate the shortest route (great circle distance) between the breeding location and the estimated average winter position. For each MDL, the derived migration distance was later used to test the relationship between migration distance and number/duration of flights/stopovers defined by the accelerometer data (see section *Statistics*) measured by the same logger. For the conventional light level geolocators we calculated the total migration distance by adding the shortest route between each estimated average stationary position and the known coordinates for the breeding site. The detour from the shortest routes was then calculated for each light level geolocator by dividing the total migration distance with the shortest route.

### Parameterization of optimization models predicting the number of flights/stopovers

Optimal migration theory is fundamentally derived from flight mechanic theory. More specifically, the flight range equation, which predicts the distance a bird can cover on a given amount of fuel [38] is a cornerstone:

1 $$Y = c\left( {1 - \frac{1}{{\sqrt {1 + f} }}} \right),$$

where *Y* is the potential flight distance, *c* is a composite coefficient with unit km that includes factors representing morphology and energy conversion efficiency, and *f* is the fuel load (as proportion of lean body mass) needed to cover distance *Y*. For the following equations we assume *c* = 15 000 km, which is a reasonable number for a shorebird [14].

To calculate the number of flights needed to cover a given distance while employing an energy minimization strategy and assuming a fixed *f*_0_, we calculate the flight distance per unit fuel according to the equation [2]:

2 $$R = \frac{{Y\left( f \right) - Y\left( {f_{0} } \right)}}{f},$$

where *Y* and *f* are defined as above (see Eq. 1), *f*_0_ is the energy cost for search/settling (*f*_0_ is proportions of lean body mass), to find optimal departure *f* (i.e. the one maximizing *R*). We maximized the equation for a range of *f*_*0*_ (0.005 to 0.1, with increments of 0.005). Subsequently, we calculated the number of flights needed to cover a distance between 500 and 3500 km, with a 100 km increment, by dividing each distance with the potential flight range (Eq. 1) for each optimal departure f^*^.

To obtain the optimal flight step length and associated number of flights for a time minimization strategy we minimized the following expression (Weber & Houston, 1997):

3 $$T\left( n \right) = n\left( {\frac{1}{k}\left( {\frac{{c^{2} }}{{\left( {c - \left( \frac{D}{n} \right)} \right)^{2} }} - 1} \right) + t_{e} } \right),$$

where *T* is the total migration time, *n* is number of flights/stopovers, *k* is the fuel deposition rate (proportion of lean body mass per day), *D* is total migration distance and *t*_*e*_ the time cost for search/settling, we calculated the optimal number of flights (*n)* for a range of *t*_*e*_ (0.1–2 days) and *k* (0.01–0.04) that minimizes the total time spent on migration.

### Statistics

The following analyses concern only data derived from the MDLs. We tested the effect of season and migration distance (zero-centered) on the number of migratory flights using generalized linear mixed models (GLMM) with a Poisson error distribution and log-linked function. We tested the effect of migration distance on total flight duration and stopover time with linear mixed models (LMM) using a Gaussian error distribution. The interaction term between season and migration distance was also included because different destinations and distances may result in different conditions along the route or at the departure site. For the GLMM we used the *glmmPQL*() function in the MASS (version 7.3-54) [39] package and for LMM the *lme*() function in R-package *nlme* (version 3.1-152) [40]. Estimated marginal means on the response variables for all mixed-models were derived using the *emmeans*() function in the R-package *emmeans*. All statistical analyses were made in R ver. 4.0.3 (http://www.r-project.org) [41].

## Results

### Characteristic route and wintering areas

**Fig. 1:**
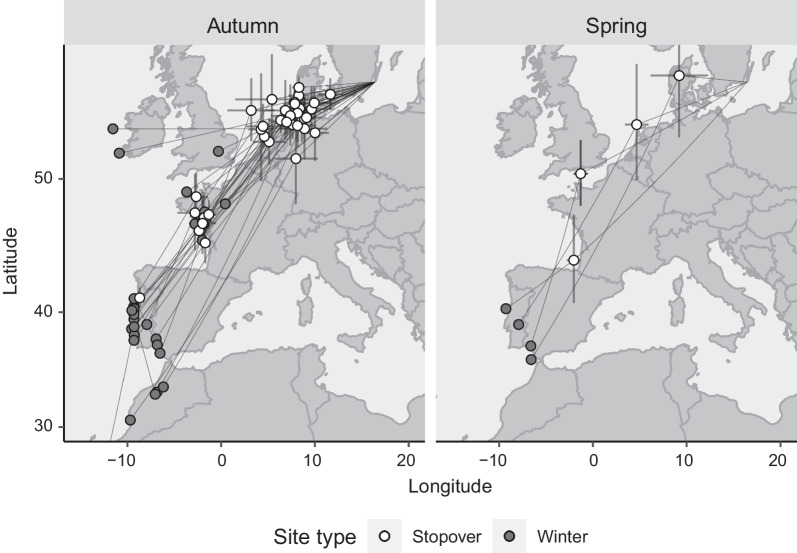
Average stopover positions (≥ 3 days; points) during autumn and spring migration (white circles) of ringed plovers breeding at Ottenby, southeast Sweden, derived from conventional light level geolocators and average wintering sites (grey circles). Error bars around stopover sites show standard deviations. For the sake of visibility error bars have been omitted from wintering sites, but can be found in Hedh et al. [28]. Lines connect sequential stationary sites of individuals. One individual wintered in west Africa and consequently has one stopover outside (south of) the displayed map. The map is a Mercator projection

**Fig. 2:**
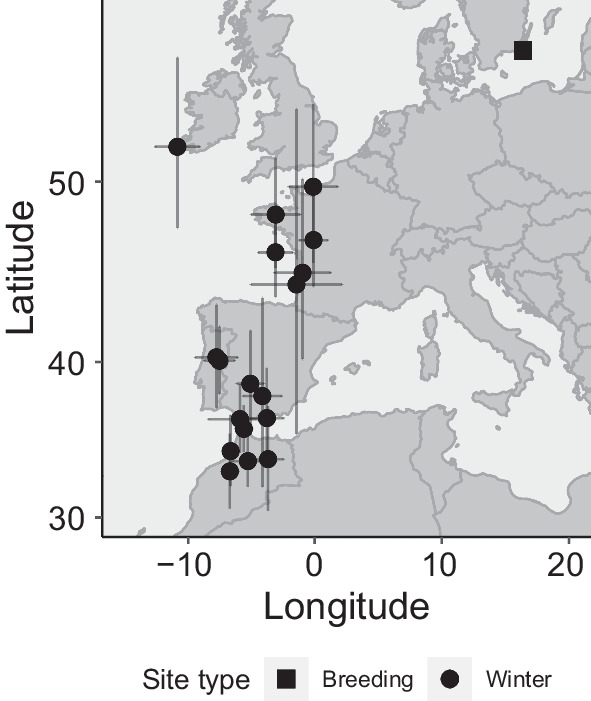
Mean wintering positions of 15 common ringed plovers breeding at Ottenby, southern Sweden based on geolocation from MDLs (see Methods). Error bars represent standard deviation. Repeated positions are included for 3 individuals. The map is a Mercator projection

Stopovers ≥ 3 days, as registered by the conventional geolocators, were located along the west European coasts (Fig. 1). In autumn most individuals stopped around the Wadden Sea and at locations in western France, particularly in the Bay of Biscay (Fig. 1). In spring, the few stopovers recorded (i.e. before or after the vernal equinoxes) were also located along the coasts (Fig. 1). Among the individuals with one or more stopovers recorded during autumn migration, the total distance calculated between breeding, stopovers and wintering sites deviated on average by 1.8% (SD = 1.7, n = 25) from the shortest distance between the breeding and wintering sites. Based on the light measurements from the MDLs, two main wintering clusters could be identified: the Iberian Peninsula/Morocco and Western France, with one exception where an individual wintered on Ireland (Fig. 2). Individual migration distances between the breeding and wintering sites ranged from 1314 to 3124 km.

### Migration performance

**Fig. 3:**
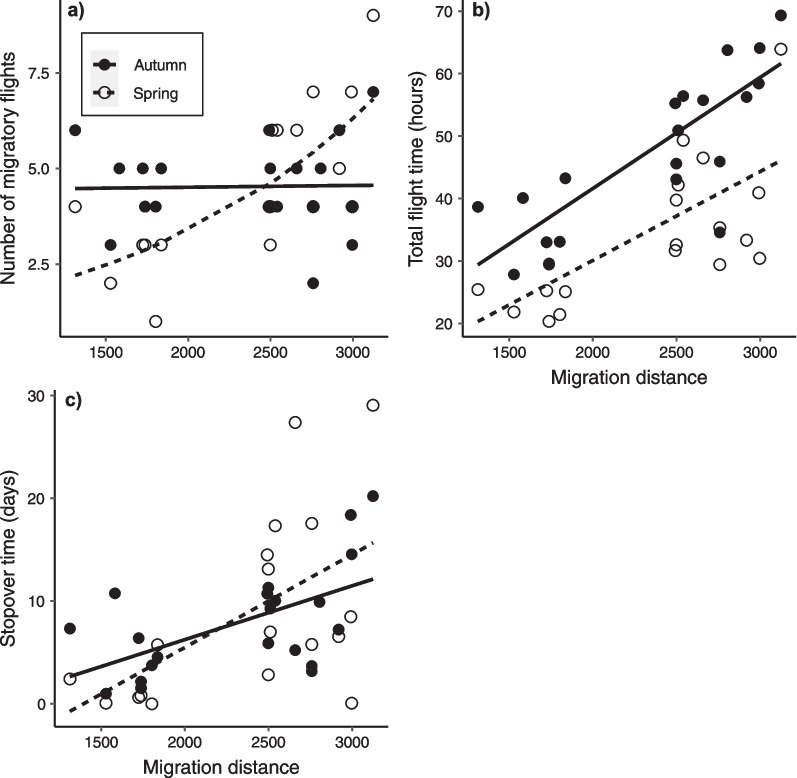
Number of **a** migratory flights, **b** total flight time (i.e. between departures and arrivals) and **c** stopover time in ringed plovers breeding at Ottenby, southeast Sweden. Filled and open circles represent individual data points from the model fit in autumn and spring, respectively. Trend lines are the linear fits between respective migration parameter and migration distance (great circle) between breeding and wintering sites. Solid lines represent autumn and dashed lines spring migration

As measured from the MDLs, there was no difference in number of flight bouts between autumn (P = 0.24; estimated marginal mean = 4.5 days, SE = 0.28, n = 21) and spring migration (4.2 days, SE = 0.28, n = 21). Nor did migration distance alone have an effect on number of flight bouts (P = 0.92). However, the interaction term between season and migration distance was significant (P < 0.001), so that there was a positive relationship between number of flights and migration distance in spring, but not in autumn (Table 1, Fig. 3a).

**Table 1:** Model output from generalized linear mixed model (GLMM) testing for differences in number of migratory flights/stopovers, as recorded by the MDLs, between seasons and total migration distance to winter site^1^

	Estimate	SE	χ^2^	*P*
*Migratory flights*				
Intercept	1.51	0.06		
Season	-0.11	0.099	1.39	0.24
Great circle distance	0.000	0.000	0.01	0.92
Season *x* great circle distance	0.000	0.000	12.96	**< 0.001**^**2**^

^1^Reference level for season is autumn

^2^Bold *P*-values denotes significant result

Over the whole migratory period, total flight time was higher in autumn (P < 0.001, Fig. 3b, Table 2; estimated marginal mean = 47.8 h, 43.6–52 95% CI, n = 21) compared to spring (35 h, 30.6–39.4 95% CI, n = 18;) and total flight time increased with increasing migration distance (P < 0.001, Fig. 3b, Table 2). Also, total stopover duration increased with migration distance (P = 0.03; Fig. 3c, Table 2). However, there was no difference between autumn (P = 0.87; 8.1 days, 5.2–11 95% CI, n = 21) and spring migration (8.6 days, 5.5–11.8 95% CI, n = 18).

**Table 2:** Estimates and SE output from linear mixed model (LMM). A type III analysis of variance (Satterthwaite’s method) was used to test for differences in flight and stopover duration (measured with MDLs) between seasons and the migration distance to winter site^1^

	n	Estimate	SE	Df	t-value	*P*
*Total flight time*						
Intercept	39	46.75	1.96	1		
Season		− 12.555	1.93	1	− 6.49	**< 0.001**^**2**^
GC distance		0.018	0.003	1	5.27	**< 0.001**^**2**^
Season *x* GC distance		− 0.004	0.0035	1	− 1.02	0.32
*Stopover duration (recorded)*						
Intercept	39	7.76	1.36	1		
Season		0.33	2.01	1	0.17	0.87
GC distance		0.005	0.002	1	2.16	0.03
Season *x* GC distance		0.004	0.004	1	1.06	0.29

^1^Reference level for season is autumn

^2^Bold P-values denotes significant result

### Individual flight and stopover durations

**Fig. 4:**
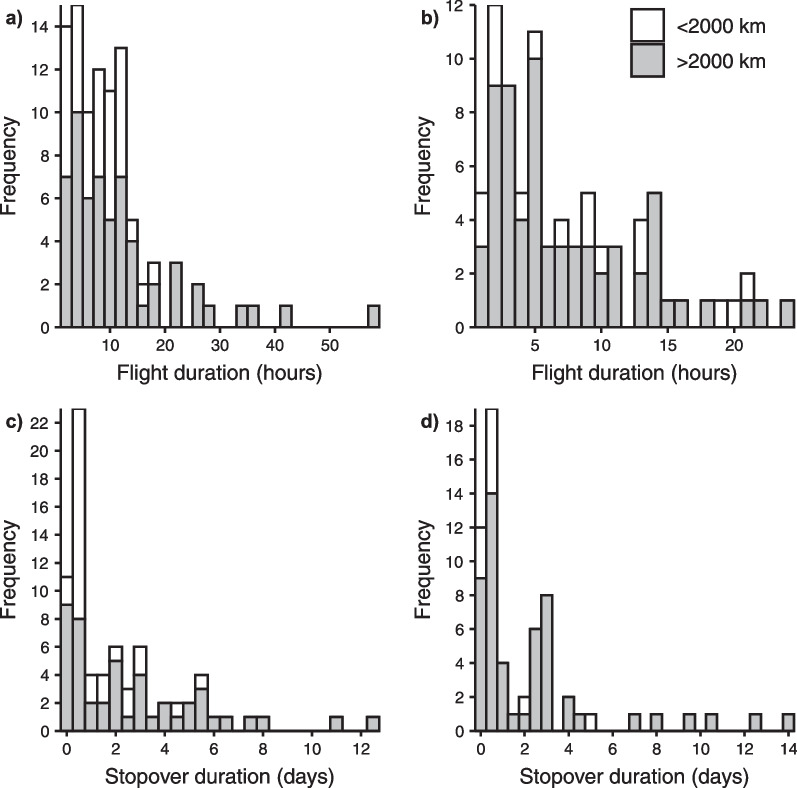
Distribution of flight duration in individual migratory flights and stopovers: **a** distribution of individual flight durations in autumn and **b** spring migration (in hours), and distribution of individual stopover duration during **c** autumn and **d** spring migration (in days). White bars represent flights and stopovers of individuals with a total migration distance < 2000 km and grey > 2000 km. One individual had a stopover lasting 23 days during spring migration but is not shown for improved visual comparison between (**c**) and (**d**)

The median duration of individual migratory flights, as measured with the MDLs, was 8.7 h in autumn (range 1.2–57.2 h, n = 95) and 5.5 h in spring (range 1–24.2 h, n = 81). In autumn, all longer flights (> 20 h) were performed by individuals migrating > 2000 km (i.e. to the Iberian Peninsula and Morocco), recorded in 9 out of 13 individuals (Fig. 4). The longest flight performed by individuals migrating < 2000 km was 17.1 h (Fig. 4a). In spring, longer flights (> 20 h) occurred only on 5 occasions, in which 2 were performed by individuals migrating < 2000 km (Fig. 4b). Among all tracks, all flights of longest duration in autumn were performed after at least one previous flight, most often as the third or fourth flight (i.e., the longest flights were never the first; see Additional file 1: Fig. S3). On the other hand, in spring, 4 out of 5 of the longer flights (> 20 h) were first flights. One of these was a direct flight performed by an individual with a total migration distance of 1802 km. The median duration of individual stopovers was 1.1 days in autumn (range 0.06–12.7 days, n = 95) and 0.8 days in spring (range 0.04–23.2 days, n = 81) (Fig. 4c, d). All longer stopovers (> 7 days) were by individuals migrating > 2000 km.

### Optimal number of flights

**Fig. 5:**
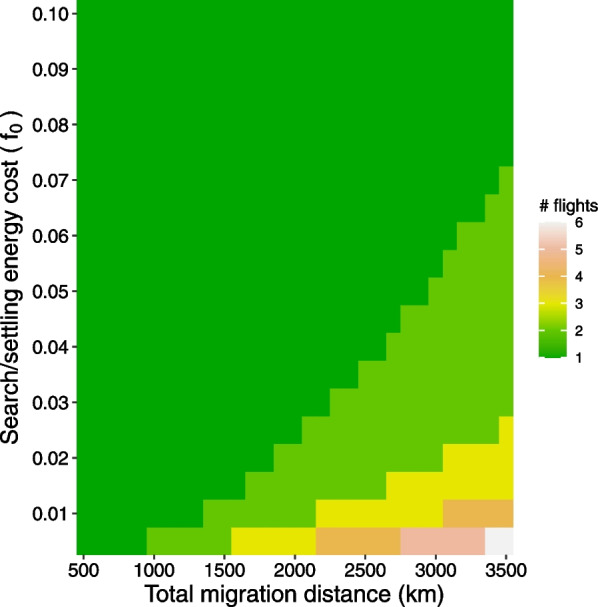
Number of flights for an energy minimizing migrant. Number of flights in relation to total migration distances and energy costs for search/settling (*f*_0_, as proportion of lean body mass), predicted according to Eq. 2, assuming that stopover sites are available everywhere along the route

**Fig. 6:**
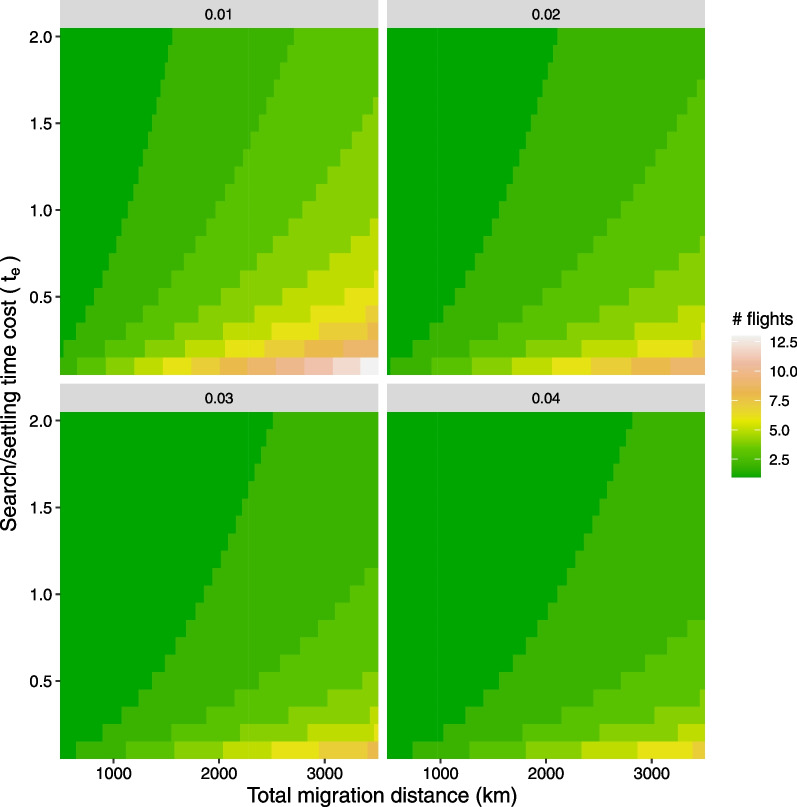
Number of flights for a time minimizing migrant. Number of flights in relation to total migration distances, fueling rates (1–4% d^−1^ of lean body mass) and time cost for search settling predicted according to Eq. 3, assuming that stopover sites are available everywhere along the route

For an energy minimizer the optimal number of flights is determined by the combination of total migration distance and the search/settling energy cost (*f*_0_) (Fig. 5), where the number of flights is 3–4 for distances around 2000 km and low. Number of flights decreases with increasing *f*_0_ and at high values the optimal policy becomes to conduct the entire migration with one or two flights (Fig. 5). For a time minimizing strategy the combination of fuel deposition rate (*k*), the search/settling time cost (*t*_e_) and migration distance determine the optimal number of flights, where low *k* (0.01) and *t*_e_ (< 0.5 days) and promotes relatively numerous (≥ 5) flights of short length (Fig. 6). For *k* = 0.02, which is close to the fueling rate at the breeding site before departure (1.7% of lean body mass per day, Hedh and Hedenström 2016), and a search/settling time cost *t*_e_ = 1 day a 3000 km migration should be divided in three flights, and if *k* = 0.03 two flights are expected (Fig. 6). With *k* = 0.04 two flights are expected for a larger range of t_e_ (≥ 0.7 days) and migration distance 3000 km (Fig. 6). A migration of 2000 km should be executed as 1–2 flights in most cased (Fig. 6).

## Discussion

According to optimal migration theory alternative strategies of energy and time minimization prescribe the disposition of the migratory journey, including where to stop along the migratory route and for how long, which depend on the environmental variables [2, 15, 16]. In this study we used both conventional light level geolocators and novel miniaturized multi sensor loggers (MDLs) to study the process of migration in a population of ringed plovers breeding in southeast Sweden. By using conventional light level geolocators we confirmed previous documented patterns of migratory routes and locations of stopovers along the European coasts, with a major stopover “hub” at the Wadden Sea [21]. Based on the MDLs we could obtain detailed information about the migration process, such as number and duration of individual flights and stopovers, not possible to obtain with conventional geolocators.

In general, the migration is performed as a sequence of several flights and stops, despite the relatively short total migration distances and relatively short duration between departure and arrival. The number of stops was similar to that of for example curlew sandpipers *Calidris ferruginea*, migrating > 10,000 km between Australia and NE Siberia [4], and those of ringed plovers migrating from Canada and northern Norway to Africa [26, 27]. However, our results deviated from the model predictions of subdividing the migration in flight episodes of equal length. A large part of this deviation is due to that in autumn nearly all individuals migrating > 2000 km (wintering on the Iberian Peninsula/Morocco) performed one flight lasting > 20 h, whereby a larger proportion of their total migration distance was covered. It is likely that birds migrating > 2000 km crossed the Bay of Biscay, because the cumulative flight time prior to the prolonged flight should have brought most individuals to western France. Thus, prolonged flights are likely to be in association with a minor barrier, the Bay of Biscay. This is noteworthy because the durations of the long flights would be enough for individuals migrating shorter distances (i.e. < 2000 km) to cover their entire migration in one flight if assuming an airspeed of 16 m s^−1^ as recorded for this species [42]. This suggests that there are either temporal or energetic limitations, as well as environmental/physiological factors, making several short flights a favourable alternative in the absence of a barrier.

Are the number of flights in accordance with those predicted by the energy- and/or time minimizing models when assuming a homogenous recourse landscape, particularly by those migrating shorter distances and in autumn? The observed number of flights tally with both models when assuming low values for fuel deposition rate *(k),* search/settling energy cost (*f*_*0*_), and search/settling time cost (*t*_e_). Indeed, field measurements of fuel deposition rates (*k*) in the ringed plover suggest relatively low population averages. In autumn at Ottenby (the breeding and first stopover site), the daily fuel accumulation rate (*k*) was estimated to 1.7% of lean body mass [43]. At Morecambe Bay, East England, (*k*) was estimated to 1.5% in spring (May) [10, 44], a value that most likely concerns birds preparing to migrate towards Greenland and Arctic Canada. If these observed fueling rates (i.e. between 1 and 2% of lean body mass per day) are typical for the whole route our observations correspond well with a time minimization strategy, where more migratory flights are predicted at low *k* (Fig. 6). Apart from the flights > 20 h, there was still a larger variation in flight duration than expected. Even though the model presented for a time minimizing strategy (Eq. 3) strictly assumes a constant fueling rate, in reality it likely varies somewhat between sites, whereby one would also expect matching responses in flight times [45], but not necessarily in number of flights [16].

Deviation from equal flight length could also be affected by other factors, such as wind conditions (head or tailwinds) that may increase or decrease flight time [8]. Some of the shorter flights may be due to unfavourable weather conditions or other reasons to interrupt a migratory flight other than for fueling [11], which could contribute to the low median stopover times in our data. Of all observed stopovers, about half lasted for a day or less, and many of these stops are likely too short to allow substantial energy accumulation as birds arriving at new stopovers generally lose mass during the first day [46]. Such mass loss may be due to physiological constraints and delayed onset of lipogenesis which will increase the values of the energetic search/settling cost (*f*_*0*_) and search/settling time cost (*t*_e_) [47]. However, remodulation of the gastrointestinal tract prior to shorter flights, which requires far less fuel stores, is probably inefficient if fueling is available with short intervals [48]. Western sandpipers (*Calidris mauri*), which travel along the American Pacific West coast and utilize several stops during spring migration [49], seem to even increase the size of digestive organs prior to the first migratory flight, suggesting that they are primed to process food at subsequent stopovers [50]. It is possible that this may be a more general pattern if feeding is available at short intervals and thus, low parameter values for both search/settling costs.

In spring however, there was a closer relationship between number of flights and migration distance than in autumn. In particular, individuals of ringed plovers migrating longer distances (> 2000 km) performed more flights, and hence had more stopovers and shorter flights in spring than in autumn. This could be explained if fueling rates decrease as the birds move towards northeast in spring (late February/early March), due to the combined effect of higher thermoregulatory costs and less food available due to low ambient temperatures, or even occasional return movements [10, 51, 52]. However, neither of these explanations clarify why ringed plovers migrating shorter distances reduce the number of flights/stopovers in spring compared to autumn.

Total flight duration was higher in autumn compared to spring, and not surprisingly, increasing with migration distance. This difference may be due to higher air speeds in spring compared to autumn. Higher air speeds in spring have been found in some nocturnal migrants and is suggested to be related to selection pressure for early arrival to the breeding sites relative to competitors [45, 53]. However, we found a ~ 35% reduction in duration of spring migration compared with autumn. Assuming that birds migrate at the maximum range speed, which minimizes the cost of transport, a 35% increase in air speed is unlikely to be sustained for longer periods of time [38]. Nilsson et al. [53] found that birds tend to increase air speed during spring migration with 14%, while Kemp et al. [54] found that supporting winds could increase spring flight speeds over Europe with up to 17% in spring compared with autumn. The combined effect of airspeed adjustment and wind support approaches the discrepancy found in this study. Another explanation could be that ringed plovers take a shorter, more direct, route in spring compared to autumn. Based on data from conventional geolocator, the calculated detour of the whole route (i.e. deviation from the closest route) was only 1.8% during autumn migration. Although a more detailed picture of the route may result in a revised estimate of the detour it should not increase much. Therefore, the most plausible explanation for shorter flight time in spring is a combination of prevailing seasonal winds and higher air speeds compared with autumn.

In conclusion, the combined use of conventional geolocators and MDLs, which record flight activity and light, generated new information about the process of migration for a temperate breeding population of ringed plover that migrates relatively short distances. We found a large variation in flight and stopover times, but the latter generally consisted of short durations. We show that ringed plovers of this population are capable of long flights (> 20 h) by which many individuals would be able to cover their entire migration distance in one flight. However, instead of doing so the birds divided the migration into several flights and stopover episodes, suggesting that shorter flights are favourable. This pattern may be more general, particularly among shorebirds migrating shorter distance and where potential stopover sites are readily available along the route.

## Supplementary Information

**Additional file 1.** Supporting figures for Methods and Results.

## Data Availability

The datasets generated and/or analysed during the current study are available in the Dryad repository 10.5061/dryad.wh70rxwr4.
