# Supplementary material for: Consequences of migratory distance, habitat distribution and season on the migratory process in a short distance migratory shorebird population
Source: Mov Ecol. 2023 Jul 18;11:40. doi: 10.1186/s40462-023-00400-6 (PMC10355079; doi:10.1186/s40462-023-00400-6)
Supplement: Supplementary file 1 — Additional file 1. Supporting figures for Methods and Results. [file 40462_2023_400_MOESM1_ESM.docx]

**Supplementary material:**

*Additional file 1*

**Figure 1.** **Sampling routine of accelerometer data collected by micro-controlled multisensory data loggers and data interpretation.** Every hour 12 activity scores are recorded between 0 and 5. One recording was done every 5 minutes consisting of 5 consecutive subsamples in 5-second intervals, each lasting 100 ms, at 100Hz. Activity at each subsample was determined if 3 out of the 10 readings deviated from the average acceleration with more than [3/g]. The sum of activity (0 or 1) at each subsample are recorded as the 5-minute activity score (0-5). We weighted the scores for each hour by using the sum of the product of each activity score and its’ hourly count. The weighted scores were subsequently used to identify flight periods.

**Figure 2. Example** **actogram derived from a ringed plover carrying a multisensor data-logger.** Behavioral data can be visualized as an actogram. Level of activity is indicated from zero (white bars) to 60 (black bars). Red bars indicate hours for which subsampling of activity had partly or fully failed. Two consecutive days have been stacked on each other (y-axis) to better visualize activity during the night. In this particular individual high activity associated with migration (dark grey to black bars) is detectable in late July and early August in autumn, and in early and late March in spring. We note that various activity patterns emerge during non-migratory periods, likely associated with lunar and tidal patterns, although these are beyond the scope of this study.


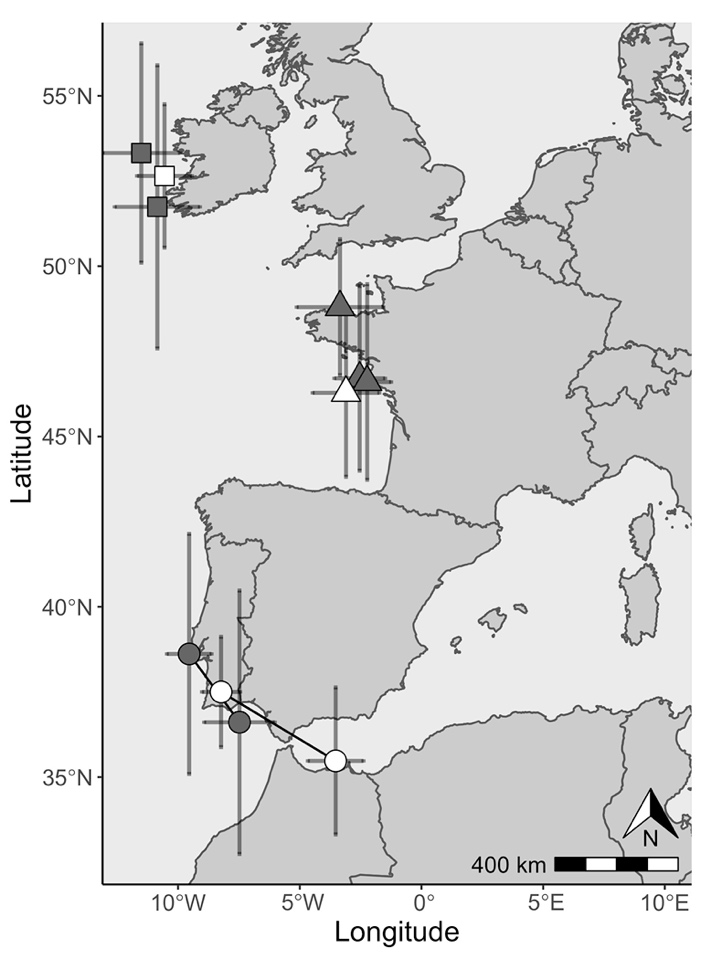


**Figure 3. Wintering positions for three individual ringed plovers estimated from conventional geolocators and multi-sensor loggers.** Consecutive wintering positions of three individual ringed plovers (symbols) estimated from different types of devices (grey symbols: conventional geolocators; white symbols: multi-sensor data loggers). Note that one individual (circles) made intra-seasonal movements, as indicated by solid lines.

**Figure 4. Distribution of flight durations across the number of migratory flights in autumn and spring.**
